# The impact of temperature on the life cycle of *Gasterophilus pecorum* in northwest China

**DOI:** 10.1186/s13071-021-04623-7

**Published:** 2021-03-01

**Authors:** Ke Zhang, Heqing Huang, Ran Zhou, Boru Zhang, Chen Wang, Make Ente, Boling Li, Dong Zhang, Kai Li

**Affiliations:** 1 Key Laboratory of Non-Invasive Research Technology for Endangered Species, School of Ecology and Nature Conservation, Beijing Forestry University, Beijing, 100083 China; 2 Chongqing Academy of Environmental Science, Chongqing, 401147 China; 3 Qinhuangdao Forestry Bureau, Qinhuangdao, 066004 Hebei China; 4 Mt. Kalamaili Ungulate Nature Reserve, Changji, 381100 Xinjiang China; 5 Xinjiang Research Centre for Breeding Przewalski’s Horse, Urumqi, 831700 Xinjiang China; 6 China National Environment Monitoring Centre, Beijing, 100012 China

**Keywords:** Desert steppe, *Gasterophilus pecorum*, Mature larvae, Population dynamics, Bimodal population, Survival rate

## Abstract

**Background:**

The departure of the mature larvae of the horse stomach bot fly from the host indicates the beginning of a new infection period. *Gasterophilus pecorum* is the dominant bot fly species in the desert steppe of the Kalamaili Nature Reserve (KNR) of northwest China as a result of its particular biological characteristics. The population dynamics of *G. pecorum* were studied to elucidate the population development of this species in the arid desert steppe.

**Methods:**

Larvae in the freshly excreted feces of tracked Przewalski’s horses (*Equus przewalskii*) were collected and recorded. The larval pupation experiments were carried out under natural conditions.

**Results:**

There was a positive correlation between the survival rate and the number of larvae expelled (*r* = 0.630, *p* < 0.01); the correlation indicated that the species had characteristic peaks of occurrence. The main periods during which mature larvae were expelled in the feces were from early April to early May (peak I) and from mid-August to early September (peak II); the larval population curve showed a sudden increase and gradual decrease at both peaks. Under the higher temperatures of peak II, the adults developing from the larvae had a higher survival rate, higher pupation rate, higher emergence rate and shorter eclosion period than those developing from peak I larvae. Although *G. pecorum* has only one generation per year, its occurrence peaked twice annually, i.e. the studied population has a bimodal distribution, which doubles parasitic pressure on the local host. This phenomenon is very rarely recorded in studies on insect life history, and especially in those on parasite epidemiology.

**Conclusion:**

The period during which *G. pecorum* larvae are naturally expelled from the host exceeds 7 months in KNR, which indicates that there is potentially a long period during which hosts can become infected with this parasite. The phenomenon of two annual peaks of larvae expelled in feces is important as it provides one explanation for the high rate of equine myiasis in KNR. 
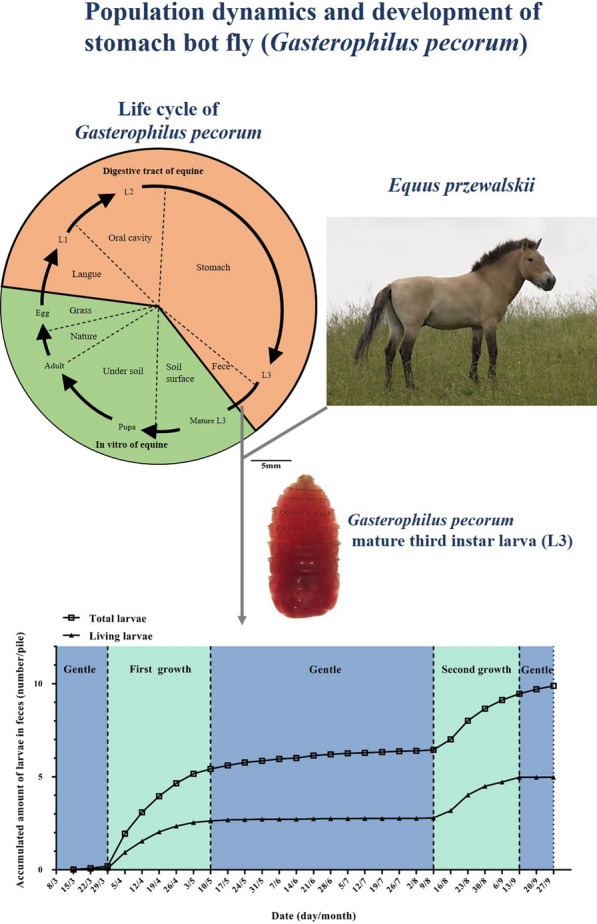

## Background

Horse stomach bot flies (*Gasterophilus* spp.) are common obligate parasites in equids [1, 2]. *Gasterophilus* larvae parasitize the digestive tract of equids and cause inflammation, ulceration, herniation and other symptoms [3–5]. The larvae absorb nutrients from the host and secrete toxins, and may lead to host death when the level of infection is severe [6, 7]. The genus *Gasterophilus* comprises 9 species globally [8, 9], of which 6 have been recorded in China [10]. The infection rate of bot flies in the equid population is 100% in Kalamaili Nature Reserve (KNR), Xinjiang, which is located in desert grassland [11]. Among the 6 species of horse stomach bot flies found in KNR, the rate of infection with *G. pecorum* is extremely high, accounting for 89–98% of all bot fly infections [11, 12]. In other regions of China, *Gasterophilus intestinalis* and *Gasterophilus nasalis* are the dominant horse stomach bot fly species [13, 14].

Horse stomach bot flies undergo complete metamorphosis; they have four developmental stages, i.e. egg, larva, pupa and adult, one generation per year, and the larvae take 9–10 months to develop in the digestive tract of the host [8]. The larvae develop in the digestive tract of equids until they are mature, then leave the host and develop into flies and start a new life cycle. The mature larva is the first stage of the host examined* in vitro*, and the population dynamics of these larvae determine subsequent population growth. The most significant feature of the life cycle of *G. pecorum* is its “unusual” reproductive strategy of laying eggs on grass [8]. Most studies on *G. pecorum* follow on from the early biological observations of Chereshnev in Kazakhstan [15], who found that the mature larvae mainly appear in early August to early September. However, a study designed to examine the development period of pupae reported a significant number of *G. pecorum* larvae collected in KNR in spring, thus the highest incidence of mature larvae was considered to be spring [16]. Based on the differences between some recent findings and existing life history records of *G. pecorum*, a systematic study of the population dynamics and growth of local *G. pecorum* was carried out* in vitro* to understand the development of this species in the desert steppe.

Insect are poikilotherms, whose metabolism, life cycle and lifespan are influenced by the ambient temperature [17, 18]. Horse stomach bot flies are exposed to a relatively stable environment in the digestive tract of the host, but once expelled from the host will be affected by the external temperature [19]. Horse stomach bot flies are thermophilic insects that tend to live at high temperatures [20]. Higher temperatures within a preferential temperature range accelerate the development rate of insects [21, 22]. In addition, the survival rate of insects metamorphosing between different states is also affected by temperature [23–25]. Knowledge of the developmental response of insects to temperature is important for an understanding of their ecology and life history [26]. Thus, we conducted an experiment to investigate the effect of different temperatures on the developmental rate, survival, pupation and emergence of *G. pecorum* to determine parameters that can be used to predict and manage horse stomach bot fly populations in KNR.

## Methods

### Study area

KNR is located in the desert subregion of northwest China (44°36′ ~ 46°00′N, 88°30′ ~ 90°03′E). KNR has an area of 18,000 km^2^, an altitude of 600–1464 m, an average annual temperature of 2.4 ℃, average annual precipitation of 159 mm and annual evaporation of 2090 mm. Winter in the desert steppe is cold and long, lasting from late October to early March [27, 28]. Protected animals in KNR include the reintroduced species *Equus przewalskii* (EN) and the endangered species *Equus hemionus* (NT), as well as *Gazella subgutturosa* (VU) and *Ovis ammon* (NT). Domestic livestock such as horses graze in KNR seasonally [29].

### Materials

Based on the climate of KNR and the life history of stomach bot flies in the area, the population survey of *G. pecorum* larvae was carried out from early March to late September 2018. The feces of Przewalski’s horses were inspected for this study. As the horses defecated one distinct pile in a single defecation event, we could count the number of piles of feces for the survey (Additional file 1). Larvae were collected from fresh feces 4–6 days/week, and the number of feces (piles of feces) and larvae were statistically analyzed on a weekly basis. We investigated fresh feces from 50–100 piles/week within 5 min after the horses defecated, and used tweezers to separate larvae from the feces. The larvae of *G. pecorum* were collected and third-instar stomach bot fly larvae identified according to Li et al.’s method [30].

1 $${\text{Proportion of feces containing larvae}}\;\left( {{\text{PF}},\% } \right)\; = \;\begin{array}{*{20}c} {\frac{{{\text{Number of feces with larvae}}}}{{{\text{Number of feces investigated}}}}\; \times \;100\% } \\ \end{array}$$

2 $${\text{Number of larvae per fece }}\left( {{\text{NL}}} \right) = \frac{{{\text{Number of larvae in feces}}}}{{{\text{Number of feces investigated}}}}$$

Transparent plastic cups (8 cm in diameter, 6 cm in height) were used as the pupation containers for *G. pecorum* larvae. Five larvae were placed in each cup, the cup mouth sealed with gauze and the larvae cultured in outdoor shade (low light intensity, where the photoperiod was that of the natural environment) in KNR. The pupation and eclosion behavior of *G. pecorum* were observed daily. The number of insects in each phase was recorded, and the survival rate, pupation rate and eclosion rate calculated. The temperature determined by thermometer at the larvae culture site was recorded at 2:00, 8:00, 14:00, 20:00 hours daily.

### Data analysis

To evaluate the survival, pupation and eclosion rates of the stomach bot flies, the following formulas were utilized:

3 $${\text{Survival rate }}\left( {{\text{SR}},\% } \right) = \frac{{{\text{Number of surviving larvae}}}}{{{\text{Total number of larvae in feces}}}} \times 100\%$$

4 $${\text{Pupation rate }}\left( {{\text{PR}},\% } \right) = \frac{{{\text{Number of pupae }}}}{{{\text{Number of surviving larvae}}}} \times 100\%$$

5 $${\text{Eclosion rate }}({\text{ER}},\% )\; = \;\frac{{{\text{Number of adults}}}}{{{\text{Number of pupae}}}}\; \times \;100\%$$

Spearman’s correlation analysis was used to analyze the relationship between the number of mature larvae and their survival rate; the significance level was set as α = 0.05. Data analysis was performed in SPSS 20.0, and the graphs were drawn by using Sigmaplot 12.0 and Graphpad prism 7.

## Results

### Population dynamics of mature larvae of *G. pecorum*

A total of 2,021 piles of equine feces were examined, of which 443 (21.92%) contained *G. pecorum* larvae (Table 1). The proportion of feces containing larvae (PF) in early April and mid-to-late August was 56.03% and 53.23%, respectively. There were two obvious larval peaks with a significant range. In May and September, the PF gradually decreased and remained low. The PF was lower than 20% during three periods: March; mid-to-late May to the end of July; and mid-to-late September.

**Table 1:** The number of piles of equine (*Equus przewalskii*) feces investigated and the proportion of feces containing *Gasterophilus pecorum* larvae (*PF*) in Kalamaili Nature Reserve (KNR) in 2018

Date	Feces investigated (N_i,_)	Feces containing larvae (n_i_)	PF n_i_/N_i_ (%)	Larvae (m_i_)	NL (mi/Ni)	Proportion of larvae (m_i_/Σm_i_) (%)
3.15	98	2	2.04	2	0.02	0.28
3.22	94	6	6.38	7	0.07	0.99
3.29	48	3	6.25	5	0.10	0.71
4.05	59	34	57.63	103	1.75	14.63
4.12	82	45	54.88	94	1.15	13.35
4.19	71	37	52.11	62	0.87	8.81
4.26	58	18	31.03	40	0.69	5.68
5.3	59	27	45.76	30	0.51	4.26
5.10	54	13	24.07	14	0.26	1.99
5.17	43	8	18.60	8	0.19	1.14
5.24	55	9	16.36	9	0.16	1.28
5.31	51	4	7.84	4	0.08	0.57
6.7	47	5	10.64	5	0.11	0.71
6.14	57	3	5.26	3	0.05	0.43
6.21	38	4	10.53	5	0.13	0.71
6.28	88	5	5.68	5	0.06	0.71
7.5	75	5	6.67	5	0.07	0.71
7.12	100	3	3.00	3	0.03	0.43
7.19	50	2	4.00	2	0.04	0.28
7.26	100	4	4.00	4	0.04	0.57
8.2	80	1	1.25	1	0.01	0.14
8.9	39	3	7.69	3	0.08	0.43
8.16	54	18	33.33	30	0.56	4.26
8.23	108	61	56.48	108	1.00	15.34
8.30	93	46	49.46	60	0.65	8.52
9.6	79	28	35.44	36	0.46	5.11
9.13	72	22	30.56	25	0.35	3.55
9.20	100	17	17.00	20	0.20	2.84
9.27	69	10	14.49	11	0.16	1.56
Total	2021	443	21.92	704	0.35	100

Values in* each row* are calculated from numbers of larvae collected between the given date and the previous date + 1 day, e.g. values for 27 September were calculated from data collected between 21 and 27 September

*NL* Number of larvae per pile of feces

**Fig. 1:**
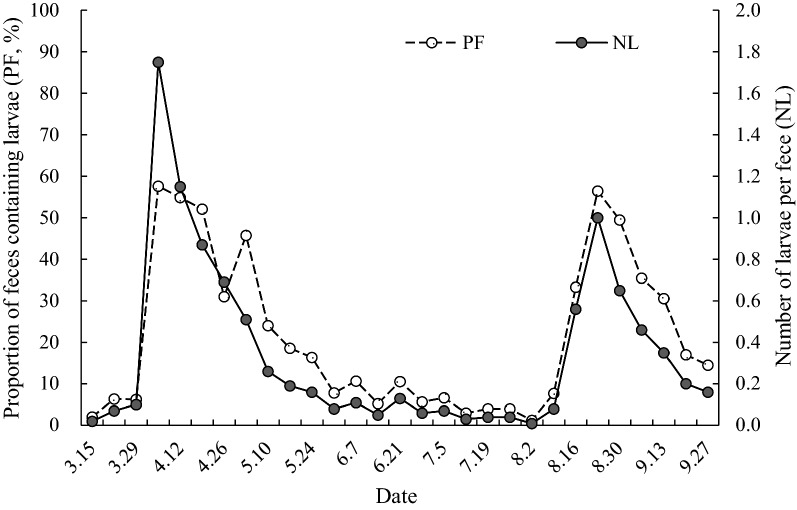
The population dynamics of *Gasterophilus pecorum* larvae in 2018 (mean/week)

A total of 704 larvae of *G. pecorum* were collected in this study. The average number of larvae per pile of feces (NL) was 0.35 during the entire investigation period; the highest number was in early April with an average of 1.40, followed by mid-to-late April and mid-to-late August, with averages of 0.79 and 0.84, respectively. The NL showed the same trend as the PF (Fig. 1).

The percentages of larvae found in April and August were 42.47% and 28.55%, respectively. The percentages of larvae were 27.98% and 14.79% in early and mid-to-late April, respectively, and 23.86% in mid-to-late August; these percentages were significantly higher than those recorded for the other periods (*p* < 0.05). The number of larvae collected from early April to early May and from mid-August to early September accounted for 48.72% and 32.52% of the total number of larvae, respectively, and the cumulative proportion of the two phases accounted for 81.24%. Thus, the main periods of mature larvae emergence in KNR were from early April to early May (peak I) and from mid-August to early September (peak II) (Fig. 1), although larvae were expelled in feces continuously throughout March to September.

The NL was derived from observations made on 10 wild horses in temporary captivity that showed that an adult Przewalski’s horse defecates an average of 10.1 piles of feces per day. It was estimated that 749 larvae of *G. pecorum* were discharged from each horse, which was close to the figure calculated from the annual average number of infective *G. pecorum* expelled locally [11].

**Fig. 2:**
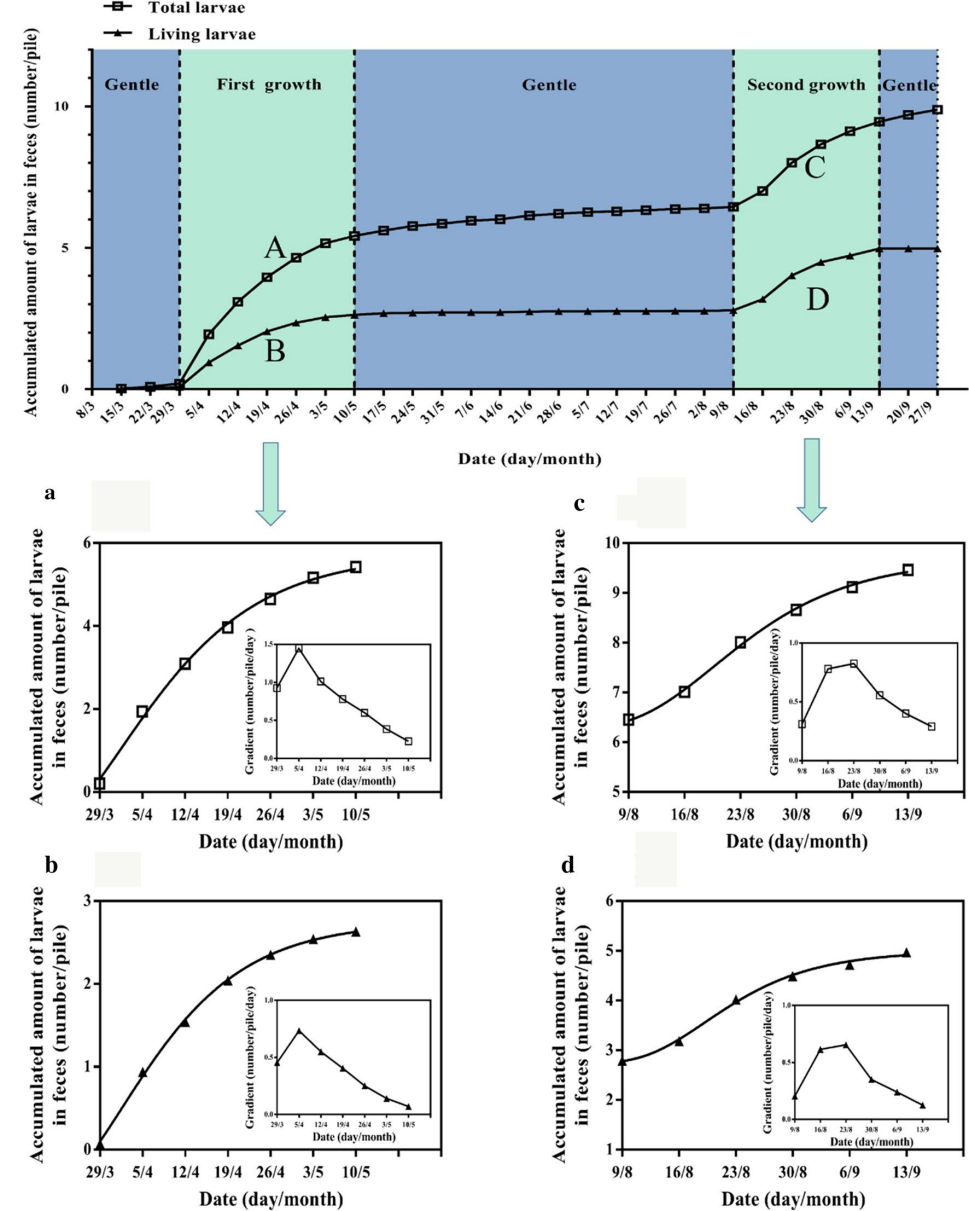
Cumulative *G. pecorum* larvae collected in 2018

The plot of the cumulative number of larvae collected during the survey period showed a double S curve (Fig. 2). There was one spike in April and one in August, and the slopes of the curves showed that in the first phase the increase in the number of total larvae (Fig. 2a) and survival of larvae (Fig. 2b) were highest on 5 April, and in the second phase (Fig. 2c, d) they were both highest on 23 August.

### Population development analysis

**Fig. 3:**
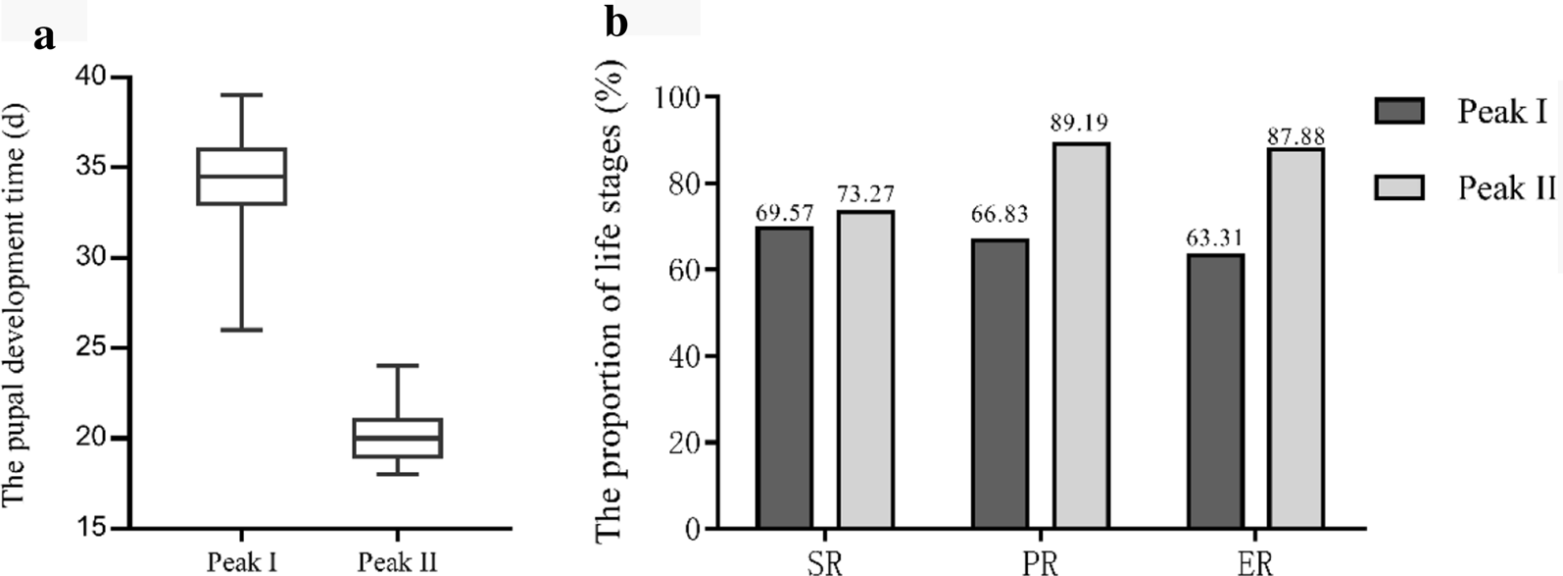
Comparison of the* in vitro* development of *G. pecorum* between the two peak periods. **a** Pupal development (days;* d*) during the two peak periods. **b** Survival rate (*SR*), pupation rate (*PR*) and eclosion rate (*ER*) of the two peak periods

The survival rates during the two peaks were 69.57% (peak I) and 73.27% (peak II) (*p* = 0.183, *t* = − 1.727), with a higher pupation rate for peak II (89.19%) than for peak I (66.83%) (*p* = 0.002, *t* = − 10.547). The eclosion rate during peak II (87.88%) was also higher than that during peak I (63.31%) (*p* = 0.002, *t* = − 9.525) (Fig. 3b).

The average pupal period of peak I and peak II lasted for 34.05 days and 20.2 days, respectively (*p* < 0.001, *t* = 15.513) (Fig. 3a). The longest and shortest development periods of peak I were 39 days and 26 days, respectively, and the longest and shortest development periods of peak II were 24 days and 18 days, respectively. There was a positive correlation between the survival rate and the number of larvae (*r* = 0.630, *p* < 0.01), i.e. the survival rate of *G. pecorum* larvae was higher during the two peak periods.

Population decreases in the three stages from the development of larvae (*N*) to adults (*N'*) was as follows: *N’* = *αβγN*, where *α* is survival rate, *β* is pupation rate and *γ* is eclosion rate. The number of adults that developed from larvae during peak I and peak II are given by *N*_1_’ and *N*_2_’, respectively; *N*_1_’ = 15.22%* N*, *N*_2_’ = 23.98%* N*.

Larvae collected during peak I that developed into adults accounted for 15.22% of the total larvae, and larvae collected in peak II that developed into adults accounted for 23.98% of the total larvae. The number of larvae collected in peak I was 1.48 times higher than that collected in peak II, but the higher survival rate, pupation rate and emergence rate of peak II resulted in 1.32 times more adults compared to peak I.

There were differences in the sex ratio between the two peak periods. The ratio of males to females in peak I was 0.73, which was lower than that in peak II (0.76) (Table 2). The proportion of female adults in relation to the total number of larvae was 0.10% in peak II, which was slightly higher than that in peak I (0.09%); this indicated that peak II was of greater significance in terms of potential *G. pecorum* infections of equids in KNR.

**Table 2:** The sex ratio of *G. pecorum* adults in the two peak periods

Period	Proportion of females (%)	Proportion of males (%)	Male to female ratio
Peak I	57.95	42.05	0.73
Peak II	43.10	56.90	1.32

Proportion of female adults that developed from the total number of larvae in peak I was 0.09%, and in peak II 0.10%

### Temperature characteristics during the periods that larva were expelled

**Fig. 4:**
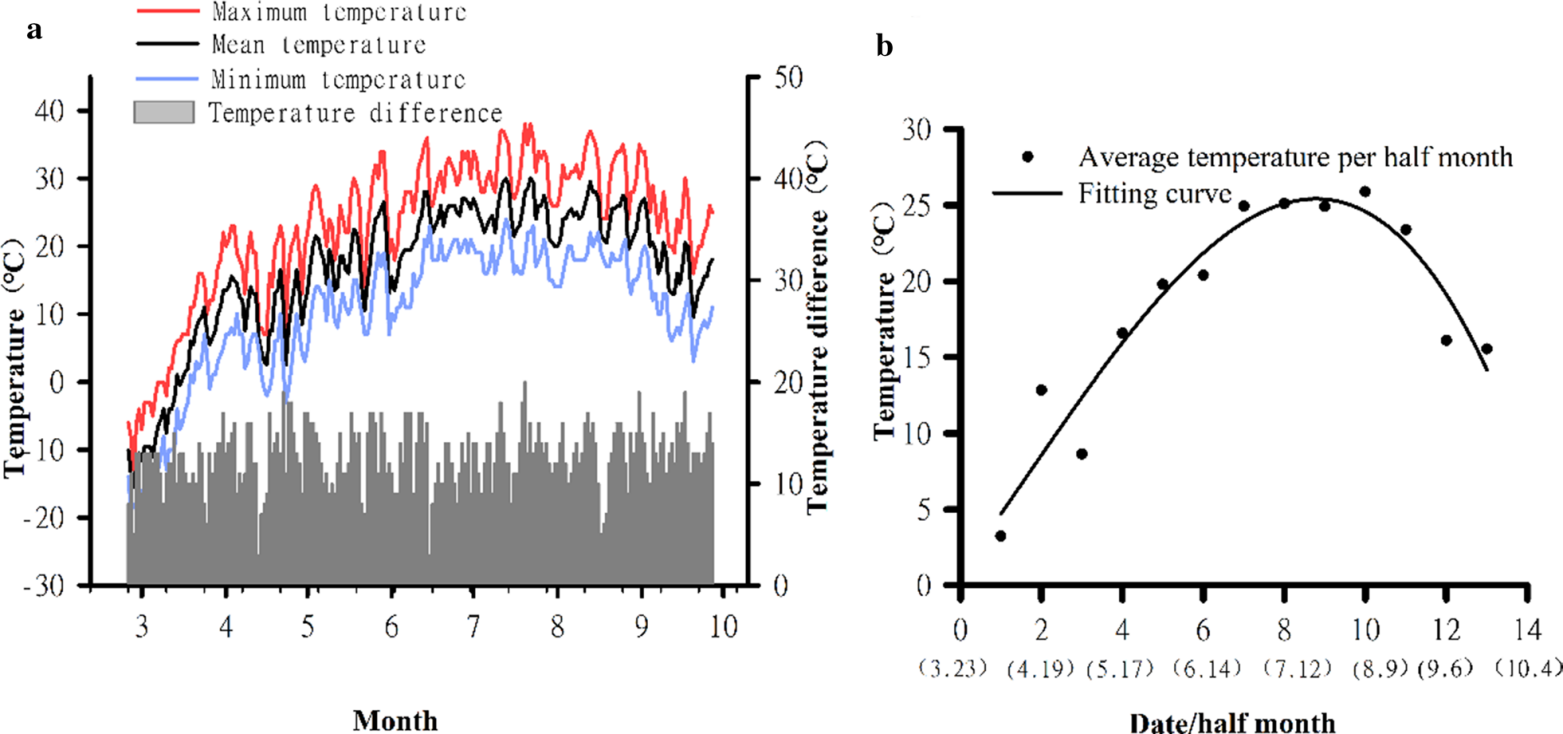
Temperature changes at KNR in 2018. **a** Average daily temperature, highest temperature, lowest temperature, and temperature difference. **b** Fitted curve

The ambient temperature rose rapidly from March to April, showed a slower increase from April to July, and began to decline after reaching the highest temperature of 38 ℃ on 20 July (Fig. 4a). The maximum daily temperature difference was 20 ℃, and the minimum and average daily temperature differences were 3 ℃ and 12.78 ℃, respectively. The temperature curve was parabolic, which is characteristic of an initially increasing then decreasing temperature (*y* = 0.8570 + 3.7449*x* + 0.1031*x*^2^ – 0.0240*x*^3^,*R*^2^ = 91.18%) (Fig. 4b). The maximum temperature, as shown by the fitted curve, was on 19 July (Fig. 4).

## Discussion

The study of insect population dynamics is an important part of insect ecological research [31, 32]. The prevalence of *G. pecorum* has been reported to be low in most countries and regions [2, 13, 33]. However, *G. pecorum* is common in the digestive tract of equids in the Mongolia-Xinjiang and Qinghai-Tibet regions of China [12, 34], central and northern Kazakhstan [14], the Republic of Mongolia [35] and the Yakut Republic of Russia [36]. There also tends to be a higher incidence of this species in certain regions. A study carried out on the period of pupal development of *G. pecorum* reported a model that can be used to predict the period of adult occurrence [16]. A study on the egg development period and the survival period of first-instar larvae of *G. pecorum* has also been completed (in submission). The results from these studies will further improve our understanding of this species and contribute to a better understanding of the epidemiology of disease caused by this parasite.

These parasites can adapt to their environment, and have different performance characteristics in different environments [37–39]. The KNR is a desert grassland with high temperatures and drought in summer, a severe and long winter with low temperatures, and low annual precipitation. The unique environmental conditions of the KNR have enabled the specific occurrence of *G. pecorum* there. These special conditions differ from those of Kazakhstan, where an earlier study of *G. pecorum* showed that the larvae were only excreted there in August [15]. The findings reported here for KNR also differ from those reported for southern Italy, where larvae of *G. pecorum* were found in January, March and July [33], and for South Africa, where adult *G. pecorum* only appeared from February to May and in August [8].

The phenology of an insect affects its development dynamics [40, 41]. The development rhythm of some insects that produce one generation per year is closely related to their phenology [42, 43]. With the change of plant growth period, the population dynamics of *Uroleucon rudbeckiae* showed a peak period of correlation [44]. In the present study, the larval population of *G. pecorum* showed two peaks annually, which were closely related to the particularly arid climate of the desert steppe. As water is the most crucial factor for life in the desert steppe [45, 46], many of the plants that grow there are ephemeral. Due to the characteristics of local precipitation [47], some *Stipa* sp. showed a special secondary growth phenomenon due to adaptation to the environment [48]. We found that the locally dominant* Stipa* began to resume growth at the end of March and early April, with seed heads developing in May, and began to grow new leaves in late August, all of which confirmed the phenomenon of secondary growth. Some studies have shown that once water conditions are suitable, many desert plants develop rapidly and have a faster life cycle [49–51]; the effects of this are reflected in the KNR ecosystem by the phenomenon of the simultaneous occurrence of *G. pecorum* and vector plants. In contrast to the other 5 species of *Gasterophilus* found in this region, which lay eggs directly on the horse host, *G. pecorum* lays eggs on* Stipa* [52]. The parasite’s population dynamics were found to match perfectly with the growth of vector plants and phenological changes, which also led to the local bimodal population distribution phenomenon of *G. pecorum* seen here. This phenomenon indicates that there are two main infection periods per year, which differs from the annual infection of animals that occurs with linear host–parasite phenomena [53, 54], and also differs from the particular characteristics of infection associated with phenology seen with most arthropod parasite infections [55–57].

In an appropriate temperature range, the higher the temperature, the more beneficial it is to the development of insects [58]. The average temperature of peak I (11.3 ± 5.3 ℃) was significantly lower than that of peak II (24.4 ± 2.7 ℃) (*t* = − 11.083, *p* < 0.001), which affected the survival rate of mature larvae. The environmental temperature during peak II was more beneficial for the subsequent pupal development stage. This resulted in a higher survival rate (survival rate, pupation rate and eclosion rate) of mature larvae from peak II, and a shorter pupal stage, which lead to the higher number of adults produced from peak II than from peak I larvae.

## Conclusions

The natural period in which *G. pecorum* larvae are expelled in horse feces in KNR exceeds 7 months. The close relationship between the bimodal population distribution of *G. pecorum* and the secondary growth of* Stipa* in the desert steppe of KNR is important as it can explain the high number of infective *G. pecorum*, high infection rate and dominance of *Gasterophilus* spp. in equids in the reserve. The larval population from peak II had a higher survival rate than that from peak I because of the suitable conditions for the development of the former. The results of this study demonstrate the highly co-evolutionary nature of the phenomenon described here in the desert steppe ecosystem, and reveal the high adaptability of organisms under adverse conditions.

## Supplementary Information

**Additional file 1: Figure S1.** Method of larvae collection and definition of piles of feces.

## Data Availability

All data generated or analyzed during this study are included in this article.

## Abbreviations

KNR: Kalamaili Nature Reserve
PF: Proportion of feces containing larvae
NL: Number of larvae per pile of feces

## References

[1] Royce LA, Rossignol PA, Kubitz ML, Burton FR (1999). Recovery of a second instar *Gasterophilus* larva in a human infant: a case report. Am J Trop Med Hyg..

[2] Mukbel R, Torgerson PR, Abo-Shehada M (2001). Seasonal variations in the abundance of *Gasterophilus* spp. larvae in donkeys in northern Jordan. Trop Anim Health Pro..

[3] Cogley TP, Cogley MC (1999). Inter-relationship between *Gasterophilus* larvae and the horse's gastric and duodenal wall with special reference to penetration. Vet Parasitol..

[4] Gökçen A, Sevgili M, Altaş MG, Camkerten I (2008). Presence of *Gasterophilus* species in Arabian horses in Sanliurfa region. Turk Soc Parasitol..

[5] Moshaverinia A, Baratpour A, Abedi V, Mohammadi-Yekta M (2016). Gasterophilosis in Turkmen horses caused by *Gasterophilus pecorum* (Diptera, Oestridae). Sci Parasitol..

[6] Smith MA, Mcgarry JW, Kelly DF, Proudman CJ (2005). *Gasterophilus pecorum* in the soft palate of a British pony. Vet Rec..

[7] Pawlas M, Sotysiak Z, Nicpoń J (2007). Existence and pathomorhological picture of gasterophilosis in horses from north-east Poland. Med Weter..

[8] Zumpt F. Myasis in man and animals in the Old World. In: Morphology, biology and pathogenesis of myiasis-producing flies in systematic order. London: Butterworths; 1965. P. 110–128.

[9] Cogley TP (1991). Key to the eggs of the equid stomach bot flies *Gasterophilus* Leach 1817 (Diptera: Gasterophilidae) utilizing scanning electron microscopy. Syst Entomol..

[10] Zhang BR, Huang HQ, Zhang D, Chu HJ, Ma XP, Li K (2018). Genetic diversity of common *Gasterophilus* spp. from distinct habitats in China. Parasites Vectors..

[11] Liu SH, Li K, Hu DF (2016). The incidence and species composition of *Gasterophilus* (Diptera, Gasterophilidae) causing equine myiasis in northern Xinjiang China. Vet Parasitol.

[12] Huang HQ, Zhang BR, Chu HJ, Zhang D, Li K (2016). *Gasterophilus* (Diptera, Gasterophilidae) infestation of equids in the Kalamaili Nature Reserve China. Parasite..

[13] Pandey VS, Ouhelli H, Verhulst A (1992). Epidemiological observations on *Gasterophilus intestinalis* and *Gasterophilus nasalis* in donkeys from Morocco. Vet Parasitol.

[14] Ibrayev B, Lider L, Bauer C (2015). *Gasterophilus* spp. infections in horses from northern and central Kazakhstan. Vet Parasitol..

[15] Chereshnev NA (1951). Biological peculiarities of the botfly *Gasterophilus pecorum* Fabr (Diptera: Gasterophilidae). Dokl Akad Nauk SSSR..

[16] Wang KH, Zhang D, Hu DF, Chu HJ, Cao J, Li K (2015). Developmental threshold temperature and effective accumulated temperature for pupae of *Gasterophilus pecorum*. Chin J Vector Biol Control..

[17] Gordon P, Harder LD, Mutch RA (1996). Development of aquatic insect eggs in relation to temperature and strategies for dealing with different thermal environments. Biol J Linn Soc..

[18] Potter K, Davidowitz G, Woods HA (2009). Insect eggs protected from high temperatures by limited homeothermy of plant leaves. J Exp Biol..

[19] Knapp FW, Sukhapesna V, Lyons ET, Drudge JH (1979). Development of third-instar *Gasterophilus* intestinalis artificially removed from the stomachs of horses. Ann Entomol Soc Am..

[20] Vincent HR, Ring TC. Encyclopedia of insects (second edition). In: Temperature, effects on development and growth. Oxford: Academic Press; 2009. p. 990–993.

[21] Ikemoto T (2015). Intrinsic optimum temperature for development of insects and mites. Environ Entomol..

[22] Wu TH, Shiao SF, Okuyama T (2015). Development of insects under fluctuating temperature: a review and case study. J Appl Entomol..

[23] Verloren MC (2010). On the comparative influence of periodicity and temperature upon the development of insects. Ecol Entomol..

[24] Roe A, Higley LG (2015). Development modeling of *Lucilia sericata* (Diptera: Calliphoridae). Peer J..

[25] Karol G, Monika F (2016). Effect of temperature treatment during development of *Osmia rufa* L. on mortality, emergence and longevity of adults. J Apic Sci..

[26] Régnière J, Powell J, Bentz B, Nealis V (2012). Effects of temperature on development, survival and reproduction of insects: experimental design, data analysis and modeling. J Insect Physiol..

[27] Zang S, Cao J, Alimujiang K, Liu SH, Zhang YJ, Hu DF (2017). Food patch particularity and forging strategy of reintroduced Przewalski’s horse in north Xinjiang China. Turk J Zool..

[28] Zhou R, Zhang K, Zhang TG, Zhou T, Chu HJ, Li K (2020). Identification of volatile components from oviposition and non-oviposition plants of *Gasterophilus pecorum* (Diptera: Gasterophilidae). Sci Rep..

[29] Liu G, Aaron BA, Zimmermann W, Hu DF, Wang WT, Chu HJ (2014). Evaluating the reintroduction project of Przewalski's horse in China using genetic and pedigree data. Biol Conserv..

[30] Li XY, Chen YO, Wang QK, Li K, Pape T, Zhang D (2018). Molecular and morphological characterization of third instar Palaearctic horse stomach bot fly larvae (Oestridae: Gasterophilinae, Gasterophilus). Vet Parasitol..

[31] Heino J, Peckarsky BL (2014). Integrating behavioral, population and large-scale approaches for understanding stream insect communities. Curr Opin Insect Sci..

[32] Modlmeier AP, Keiser CN, Wright CM, Lichtenstein JLL, Pruitt JN (2015). Integrating animal personality into insect population and community ecology. Curr Opin Insect Sci..

[33] Otranto D, Milillo P, Capelli G, Colwell DD (2005). Species composition of *Gasterophilus* spp. (Diptera, Oestridae) causing equine gastric myiasis in southern Italy: parasite biodiversity and risks for extinction. Vet Parasitol..

[34] Wang WT, Xiao S, Huang HQ, Li K, Zhang D, Chu HJ (2016). Diversity and infection of *Gasterophilus* spp. in Mongol-Xinjiang region and Qinghai Tibet region. Sci Silva Sin..

[35] Dorzh C, Minár J (1971). Warble flies of the families Oestridae and Gasterophilidae (Diptera) found in the Mongolian People's Republic. Folia Parasit..

[36] Reshetnikov AD, Barashkova AI, Prokopyev ZS (2014). Infestation of horses by the causative agents of gasterophilosis (Diptera: Gasterophilidae): the species composition and the north-eastern border of the area in the Republic (Sakha) of Yakutia of the Russian Federation. Life Sci J..

[37] Gandon S, Ebert D, Olivieri I, Michalakis Y, Mopper S, Strauss SY (1998). Differential adaptation in spacially heterogeneous environments and host-parasite coevolution. Genetic structure and local adaptation in natural insect populations.

[38] Thomas F, Renaud F, Guégan JF. Parasitism and ecosystems. In: Parasitism and hostile environments. New York: Oxford University Press; 2015. p. 85–112.

[39] Machado TO, Braganca MAL, Carvalho ML, Andrade FJD (2012). Species diversity of sandflies (Diptera: Psychodidae) during different seasons and in different environments in the district of Taquaruçú, state of Tocantins, Brazil. Mem I Oswaldo Cruz..

[40] Aliakbarpour H, Che SMR, Dieng H (2010). Species composition and population dynamics of thrips (Thysanoptera) in mango orchards of Northern Peninsular Malaysia. Environ Entomol..

[41] Palomo LAT, Martinez NB, Napoles JR, Leon OS, Arroyo HS, Graziano JV (2015). Population fluctuations of thrips (Thysanoptera) and their relationship to the phenology of vegetable crops in the central region of Mexico. Fla Entomol..

[42] Shibata E (2008). Seasonal fluctuation and spatial pattern of the adult population of the Japanese pine sawyer, *Monochamus alternatus* Hope (Coleoptera: Cerambycidae), in young pine forests. Appl Entomol Zool..

[43] Haack RA, Lawrence RK, Heaton GC (2018). Seasonal shoot-feeding by *Tomicus piniperda* (Coleoptera: Scolytidae) in Michigan. Great Lakes Entomol..

[44] Lamb RJ, Mackay PA (2016). Seasonal dynamics of a population of the aphid *Uroleucon rudbeckiae* (Hemiptera: Aphididae): implications for population regulation. Can Entomol..

[45] Huxman TE, Smith MD, Fay PA, Knapp AK, Shaw MR, Loik ME (2004). Convergence across biomes to a common rain-use efficiency. Nature.

[46] Cleland EE, Collins ST, Dickson TL, Farrer EC, Gross KL, Gherardi LA (2013). Sensitivity of grassland plant community composition to spatial vs. temporal variation in precipitation. Ecology.

[47] Yong SP, Zhu ZY (1992). A certain fundamental characteristics of Gobi Desert vegetation in the centre Asia. Acta Sci Natl Univ Neimongol..

[48] Cui NR. The flora records of main forage grass crops in Xinjiang. In: Book one. Urumqi: Xinjiang People’s Publishing House; 1990. p. 140–157 (**in Chinese**).

[49] Ogle K, Reynolds JF (2004). Plant responses to precipitation in desert ecosystems: integrating functional types, pulses, thresholds, and delays. Oecologia.

[50] Mckenna MF, Houle G (2010). Why are annual plants rarely spring ephemerals?. New Phytol..

[51] Tielbörger K, Valleriani A (2010). Can seeds predict their future? Germination strategies of density-regulated desert annuals. Oikos.

[52] Liu SH, Hu DF, Li K (2015). Oviposition site selection by *Gasterophilus pecorum* (Diptera: Gasterophilidae) in its habitat in Kalamaili nature reserve, Xinjiang China. Parasite..

[53] Epe C, Kings M, Stoye M, Böer M (2001). The prevalence and transmission to exotic equids (*Equus quagga antiquorum*, *Equus przewalskII*, *Equus africanus*) of intestinal nematodes in contaminated pasture in two wild animal parks. J Zoo Wildl Med..

[54] Hu XL, Liu G, Zhang TX, Yang S, Hu DF, Liu SQ (2018). Regional and seasonal effects on the gastrointestinal parasitism of captive forest musk deer. Acta Trop..

[55] Teel PD, Marin SL, Grant WE (1996). Simulation of host-parasite-landscape interactions: influence of season and habitat on cattle fever tick (*Boophilus* sp.) population dynamics. J Am Soc Nephrol..

[56] James PJ, Moon RD, Brown DR (1998). Seasonal dynamics and variation among sheep in densities of the sheep biting louse *Bovicola ovis*. Int J Parasitol..

[57] Taylor B, Rahman PM, Murphy ST, Sudheendrakumar VV (2012). Within-season dynamics of red palm mite (*Raoiella indica*) and phytoseiid predators on two host palm species in south-west India. Exp Appl Acarol..

[58] Hercus MJ, Loeschcke V, Rattan SIS (2003). Lifespan extension of *Drosophila melanogaster* through hormesis by repeated mild heat stress. Biogerontology.

