# Supplementary material for: The impact of temperature on the life cycle of Gasterophilus pecorum in northwest China
Source: Parasit Vectors. 2021 Mar 1;14:129. doi: 10.1186/s13071-021-04623-7 (PMC7923332; doi:10.1186/s13071-021-04623-7)

Przewalski's horses will leave one defined 'pile' in a single defecation event.


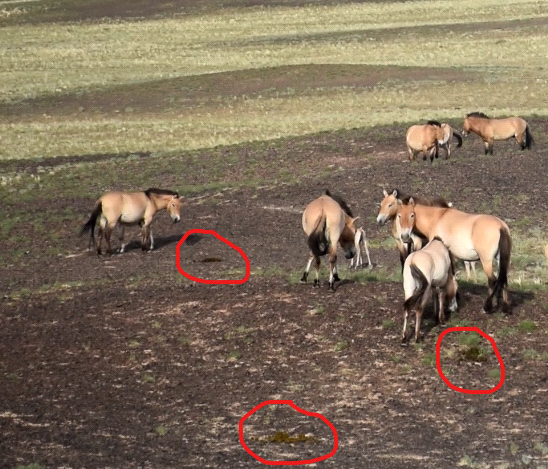


We used tweezers to break up fresh feces and separated the larvae from the feces.


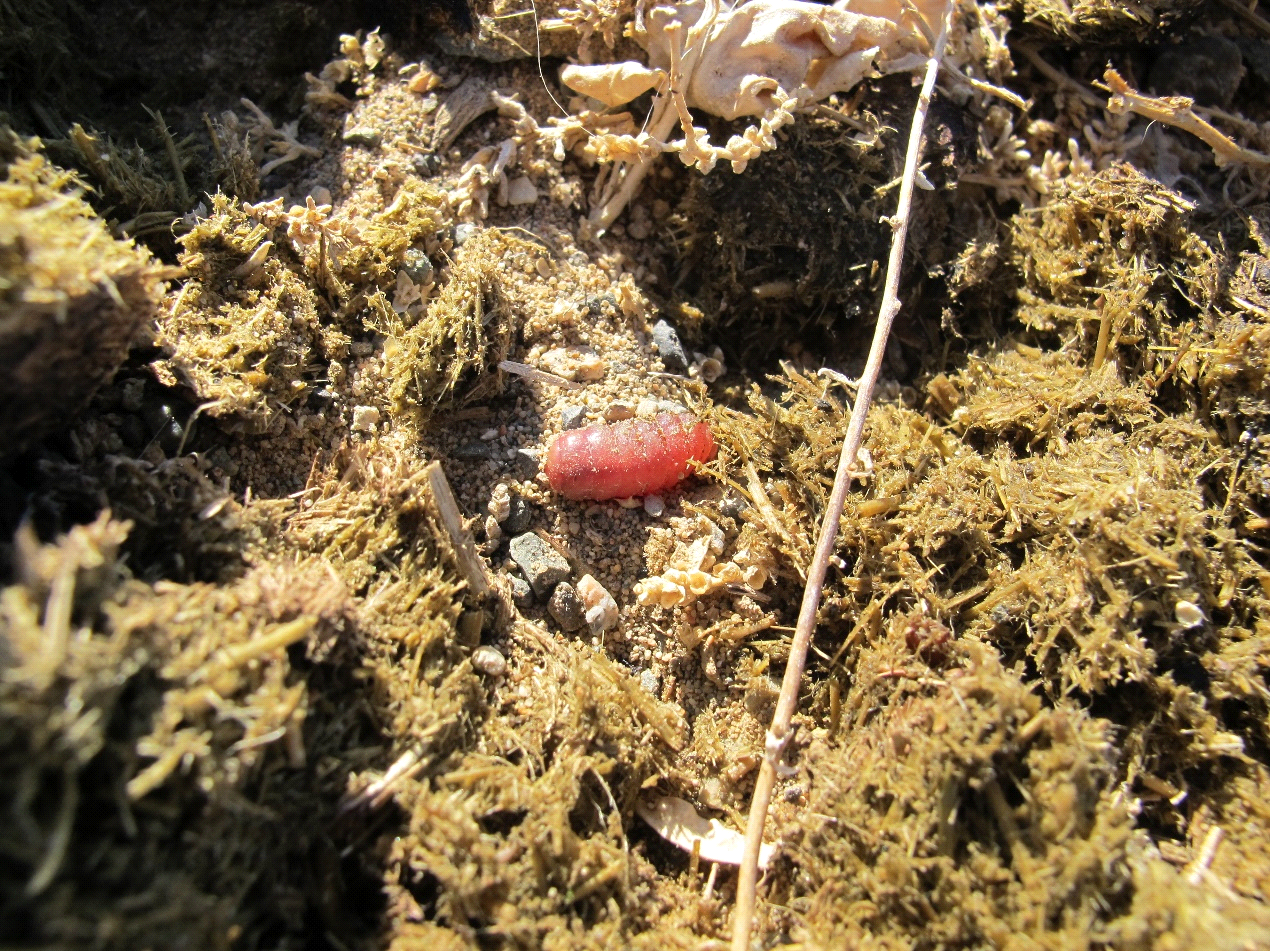

Supplement: Supplementary file 1 — Additional file 1: Figure S1. Method of larvae collection and definition of piles of feces. [file 13071_2021_4623_MOESM1_ESM.docx]
